# Pediatric Exposure to Drugs of Abuse by Hair Testing: Monitoring 15 Years of Evolution in Spain

**DOI:** 10.3390/ijerph110808267

**Published:** 2014-08-14

**Authors:** Simona Pichini, Oscar García-Algar, Airam-Tenesor Alvarez, Maria Mercadal, Claudia Mortali, Massimo Gottardi, Fiorenza Svaizer, Roberta Pacifici

**Affiliations:** 1 Drug Abuse and Doping Unit, Department of Therapeutic Research and Medicines Evaluation National Institute of Health, Roma 00161 Italy; E-Mails: claudia.mortali@iss.it (C.M.); roberta.pacifici@iss.it (R.P.); 2 Unitat de Recerca Infància i Entorn (URIE), Paediatric Service, Institut de Recerca Hospital del Mar—IMIM, Barcelona 80003, Spain; E-Mails: 90458@hospitaldelmar.cat (O.G.-A.); 60809@hospitaldelmar.cat (A.-T.A.); 60810@hospitaldelmar.cat (M.M.); 3 Red SAMID, RETICs, Instituto de Salud Carlos III, Madrid 28002, Spain; 4 Laboratorio di Sanità Pubblica (LSP), Azienda Provinciale Servizi Sanitari, Trento 38121, Italy; E-Mails: massimo.gottardi@comedicaltrento.it (M.G.); fiorenza.svaizer@apss.tn.it (F.S.)

**Keywords:** hair testing, drugs of abuse exposure, children, Spain

## Abstract

Hair testing is a useful tool to investigate the prevalence of unsuspected chronic exposure to drugs of abuse in pediatric populations and it has been applied to three different cohorts of children from Barcelona, Spain along fifteen years to evaluate eventual changes in this exposure. Children were recruited from three independent studies performed at Hospital del Mar (Barcelona, Spain) and approved by the local Ethics Committee. Hair samples were collected from the first 187 children cohort (around 4 years of age) in 1998, from the second 90 children cohort (1.5–5 years of age) in 2008 and from the third 114 children cohort (5–14 years of age) in 2013. Hair samples were analysed for the presence of opiates, cocaine, amphetamines, and cannabis by validated methodologies using gas or liquid chromatography-mass spectrometry. Familiar sociodemographics and eventual consumption of drugs of abuse by parents, and caregivers were recorded. Hair samples from 24.6% children in 1998 were positive for any drug of abuse (23.0% cocaine), 25.5% in 2008 (23.3% cocaine), and 28.1% in 2013 (20.1% cocaine and 11.4% cannabis). In none of the cohorts, parental sociodemographics were associated with children exposure to drugs of abuse. The results of the three study cohorts demonstrated a significant prevalence of unsuspected pediatric exposure to drugs of abuse which mainly involved cocaine maintained along fifteen years in Barcelona, Spain. We recommend to be aware about unsuspected passive exposure to drugs of abuse in general population and to use general or selected hair screening to disclose exposure to drugs of abuse in children from risky environments to provide the basis for specific social and health interventions.

## 1. Introduction

Since the second half of the 90s, two bi-yearly surveys concerning use of legal and illegal psychoactive drugs based on self-filled questionnaires have been carried out in Spain: the ETADES [1,2] survey on tobacco, alcohol and drugs of abuse consumption in the general population (14–65 years of age) and ESTUDES survey [3,4] concerning use of above reported substances in secondary school students.

According to the data reported in the survey on general population, between 1999 and 2011 the consumption of drugs of abuse in the previous 12 months remained stable for the majority of the substances (alcohol: 74.6% *vs.* 76.6%; tobacco: 43.7% *vs.* 40.2%, MDMA: 0.8% *vs.* 0.7%, amphetamines: 0.7% *vs.* 0.6%, hallucinogens: 0.6% *vs.* 0.4% and heroin 0.1% *vs.* 0.1%), with a significant increase in cannabis and cocaine consumption (6.8% *vs.* 9.6% and 1.5% *vs.* 2.3%), respectively [1,2].

These figures place Spain in the first place among the European countries concerning cocaine and cannabis consumption in the general population, exceeding even those reported from America [3]. Concerns regarding the impact of these high levels of cannabis and cocaine use in Spain on passive exposure to these drugs in pediatric population prompted the investigation on the evolution in passive exposure in children of different ages from Barcelona Spain to show the usefulness of hair testing as a tool to disclose this exposure in order to raise the public awareness on this problem and to stimulate a political debate. Hair testing results available from two investigations performed in 2009 and 2013 on two different cohorts of children [4,5] were compared with those obtained for the purpose of this study in hair samples collected in 1998 from a cohort of children of 4 years of age at that time.

## 2. Methods

### 2.1. Subjects and Samples

The investigation was carried out on non-selected (general population) children from three independent studies conducted at different times in the same setting: Hospital del Mar in Barcelona (Spain). The hospital is located in an urban area with low socioeconomic status and a high percentage (more than 40%) of immigrants [4,5]. Hair samples, cut at the scalp, were collected from all the children of the three studies. Since hair grows at a rate of approximately 1 cm/month, in order to document a possible repeated exposure, a minimum 4 cm hair strand was required.

The study was approved by the Institutional Ethics Committee, conducted in accordance with the Declaration of Helsinki and signed informed consent was obtained from the accompanying parents. Parental sociodemographics and possible toxic habits were recorded using a standard structured questionnaire usually applied at the hospital and in our previous investigations [6].

### 2.2. Study 1

Children (187, around 4 years of age) were recruited in 1998 from the Asthma Multicenter Infant Cohort Study (AMICS) [7]. The AMICS study was designed to investigate the effects of several pre- and post-natal environmental exposures on the inception of atopy and asthma. Several biological matrices from this cohort were collected from the neonatal period to 8 years of age. In particular, at 4 years of age hair samples were collected and stored for eventual analysis of environmental exposure to tobacco smoke and other toxic substances.

### 2.3. Study 2

Pre-school children (90, 1.5–5 years of age) were recruited in 2008 after being admitted to the Emergency Department for a variety of general medical complaints. Hair samples were collected and stored to investigate the prevalence of unsuspected exposure to cocaine [4]. Nonetheless, analysis for all the principal drugs of abuse were performed. Children younger than 1.5 years were excluded to avoid the possibility that the presence of drugs of abuse in hair samples could be partly due to prenatal exposure.

### 2.4. Study 3

Children (114, 2 to 10 years of age) were recruited in 2013 from a scenario similar to that of *Study 2* after being admitted to the Emergency Department for a variety of general medical complaints [5]. This time both pre-school and primary school children were considered, excluding children older than 10 years, to avoid any possibility of active drug consumers.

### 2.5. Hair Samples Analysis

All the hair samples were collected and stored in closed paper envelopes at dry ambient temperature until analysis. Hair samples from children belonging to *Study 2* were examined for the presence of opiates, cocaine, cannabinoids and amphetamines by a validated gas chromatography-mass spectrometry method with a limit of quantification of 0.2 ng/mg hair for all the analytes under investigation [4].

Hair samples from children belonging to the *Studies 1* and *3* were both recently examined for the presence of opiates, cocaine, cannabinoids and amphetamines by a validated ultra-performance liquid chromatography-tandem mass spectrometry methodology with a limit of quantification of 0.1 ng/mg hair for amphetamines, opiates and cocaine; 0.05 ng/mg hair for benzoylecgonine and cannabinoids [8].

### 2.6. Statistical Analysis

Statistical analysis for the continuous variables was done with the Student’s *t*-test for two groups and the analysis of variance (ANOVA) for three or more groups. The Fisher’s exact test was used for the comparison of categorical data. For the analysis of individual percentages of exposed *vs.* unexposed children, the binomial test was used. Statistical significance was set at *p* < 0.05. The SPSS program (version 22.0, SPSS Inc, Chicago, IL, USA) was used for the analysis of data.

## 3. Results

As reported in Table 1, of the 187 children of the 1998 *Study 1* cohort, 43 had hair samples positive for cocaine (median value: 0.32 ng/mg hair) and its metabolite benzoylecgonine (median value: 0.26 ng/mg hair) (23.0%), one was positive for 6-monoacetylmorphine and morphine (0.30 ng/mg and 0.25 ng/mg hair, respectively) and three to codeine (median value 0.44 ng/mg hair) for a total of 24.6% children exposed to drugs of abuse. No hair samples resulted positive to Δ9-tetrahydrocannabinol. Similarly, of the 90 children of the 2008 *Study 2* cohort, 21 had hair samples positive for cocaine (median value 1.57 ng/mg hair) and its metabolite (median value of 0.85 ng/mg hair) (23.3%), with one sample positive for 6-monoacetylmorphine and morphine (0.35 ng/mg and 0.21 ng/mg hair, respectively) and one to 3,4-methylenedioxymethamphetamine (MDMA) (0.65 ng/mg hair) and its metabolite 3,4-methylenedioxyamphetamine (MDA) (0.59 ng/mg hair) for a total of 25.5% exposed children. Also in this cohort, no child indicated exposure to cannabis products. Conversely, of the 114 children hair samples of the 2013 *Study 3* cohort, 13 had samples positive for Δ9-tetrahydrocannabinol (median value: 0.16 ng/mg hair) and cannabidiol (median value: 0.15 ng/mg hair) with one sample also positive for cannabinol (0.79 ng/mg hair) too (11.4%). Cocaine (median value: 0.54 ng/mg hair) and benzoylecgonine (median value: 0.18 ng/mg hair) were found in 23 hair samples (20.1%) with 6-MAM (0.42 ng/mg hair) and morphine (0.15 ng/mg hair), four samples positive for codeine (median value 0.17 ng/mg hair) and one sample positive for methadone (2.09 ng/mg hair) for a total of 28.1% children exposed to any drugs of abuse.

**Table 1:** Exposure to drugs of abuse in the children of the three study cohorts according to hair analysis.

	Study 1 Cohort, 1998 ^§^ (187 Children, 4 Years of Age)	Study 2 Cohort, 2008 ^§§^ (90 Children, 1.5 to 5 Years of Age)	Study 3 Cohort, 2013 ^§^ (114 Children, 2 to 10 Years of Age)
Any drug of abuse	24.6%	25.5%	28.1%
Cocaine	23.0%	23.3%	20.1%
Cannabis	ND ^*^	ND *	11.4%

^*^ ND: not detected; ^§^ limit of quantification of 0.1 ng/mg hair for amphetamines, opiates and cocaine; 0.05 ng/mg hair for benzoylecgonine and cannabinoids; ^§§^ limit of quantification of 0.2 ng/mg hair for all the analytes under investigation.

Table 2 shows the parental sociodemographic characteristics of the three study cohorts considering non-exposed and exposed children separately.

**Table 2:** Parental sociodemographics and exposure to drugs of abuse for each cohort children according to hair analysis.

	Study 1 Cohort, 1998 (187 Children, 4 Years of Age)		Study 2 Cohort, 2008 (90 Children, 1.5 to 5 Years of Age)		Study 3 Cohort, 2013 (114 Children, 2 to 10 Years of Age)	
	Children Not Exposed to Any Drug of Abuse (*n* = 141)	Children Exposed to Any Drug of Abuse (*n* = 46)	Children Not Exposed to Any Drug of Abuse (*n* = 67)	Children Exposed to Any Drug of Abuse (*n* = 23)	Children Not Exposed to Any Drug of Abuse (*n* = 82)	Children Exposed to Any Drug of Abuse (*n* = 32)
**Mother’s nationality (%)**						
Spanish	68.8	78.3	59.6	75.0	57.5	72.0
Non Spanish	31.2	21.7	40.4 ^*^	25.0	42.5 ^*^	28.0 ^*^
**Father’s nationality (%)**						
Spanish	69.1	75.4	60.3	68.4	60.2	65.4
Non Spanish	30.9	24.6	39.7 ^*^	31.6 ^*^	39.8 ^*^	34.6 ^*^
**Employed mother (%)**						
No	48.8	53.4	25.4 ^*^	45.0 ^*^	32.4 ^*^	35.0 ^*^
**Mother’s profession (%)**						
Managerial, Professional & Skilled (non-manual)	22.6	18.9	18.5	10.0	8.5 ^*^	7.7 ^*^
Skilled (manual) & Partly skilled	30.5	30.8	40.0	30.0	40.3 ^*^	42.3 ^*^
Unskilled	46.9	50.3	41.5	60.0 ^*^	51.2	50.0
**Employed Father (%)**						
No	8.1	8.3	0.0	0.0	2.3 ^*^	3.4 ^*^
**Father’s profession (%)**						
Managerial, Professional & Skilled (non-manual)	24.1	15.8	20.6	10.5	10.6 ^*^	10.5
Skilled (manual) & Partly skilled	25.6	26.6	28.6	31.7	25.9	30.8
Unskilled	50.3	57.6	50.8	57.8	63.5 ^*^	58.7

^*^: *p* < 0.05 in comparison to Study 1 cohort.

Although within the three cohorts parental nationality and socio-economic status were not associated with exposure to drug in children, the percentage of foreign people increased significantly along the years, and practically doubled in those whose children were not exposed to drugs of abuse. A surprising increase in employed mothers and fathers was also noted with the passing of the years, even if this mostly concerned partly skilled and unskilled jobs.

## 4. Discussion

The most important result of this investigation, based on hair testing for drugs of abuse in children, is the consistent unsuspected high prevalence of pediatric exposure to psychotropic drugs during the last 15 years in Barcelona, Spain. Even though the age of the children was different in the three study cohorts, the percentage of exposed children remained around 25% for ten years, with a raise up to 28% in the more recent past. Apparently, this is due to the appearance of passive exposure to cannabis products in 2013, which was not observed in 1998 nor in 2008.

It has to be said that in 2008, the limit of quantification for the gas chromatographic-mass spectrometric assay available at that time, was of 0.2 ng/mg hair for all the analytes under investigation, including Δ9-tetrahydrocannabinol, the principal hair biomarker of exposure to cannabis products. Most probably, this value was not sensitive enough to disclose eventual exposure to cannabis products and this has been a study limitation at that time. Indeed, the Society of Hair Testing recommended a confirmatory cut-off of 0.05 ng Δ9-tetrahydrocannabinol per mg of hair [9]. However, when hair samples from the 1998 *Study 1* cohort were recently examined applying the limit of quantification of 0.05 ng/mg hair, no positive samples were found, showing that at least in 1998 none of the children of study cohort was exposed to cannabis product. This occurrence cannot be attributed to eventual degradation of Δ9-tetrahydrocannabinol in hair samples, which was indeed never observed. Conversely, what it can be said is that in the last 15 years cannabis consumption has increased significantly in Spain with a diffused “social acceptance” of cannabis consumption [2].

Nonetheless, it has to be recognized that the limits of quantification of 0.05 ng/mg could be still high enough to realistically disclose pediatric passive exposure to cannabinoids, particularly for older children who are not in such close contact with the consuming parents. Indeed, this observation suggests that the obtained results could have been conservative and that percentages of pediatric exposure to psychotropic drugs could have been even higher. Overlapping percentages of passive exposure to drugs in children of different age (infants, pre-school and primary school children) evidences that the phenomenon covers all the first childhood (up to 10 years) with no impact of familiar socioeconomic status or nationality, even if the examined children all come from a population of general low socio-economic status for the European standards. What was also already reported for *Studies 2* and *3* is that the first source of paediatric exposure to drugs of abuse is the parent, in the majority the cases the mother, and more in general a risky parental environment or contaminated surroundings. Indeed, hair concentrations of cocaine and benzoylecgonine were higher in younger children from *Study 2* more exposed than elder ones because of closer contact to the consuming adults, as previously demonstrated [10].

While it has been proved that acute exposure to cocaine during childhood is associated with neurologic manifestations such as focal and generalized seizures in children and toddlers and alterations in mental status, including delirium stupor and coma in older children [11,12], the long term effects of a repeated postnatal exposure to cocaine have not been clarified yet. Similarly, whereas acute exposure to cannabis in childhood was reported to cause developmental delay, hyperactivity, lethargy ataxia, and respiratory insufficiency [13,14], no information on the effects of chronic pediatric exposure to that drug is known. Nevertheless, even if it is difficult to identify a core set of physical, cognitive, or behavioural problems that can be directly attributed to postnatal (or pre- and postnatal) exposure to drugs of abuse, it has been widely demonstrated that paediatric exposure to drugs of abuse is frequently associated with poor child care by the care giver. In this concern, *Studies 2* and *3* showed that parents of children exposed to drugs of abuse present, to a significantly higher extent, behavioural patterns with potential harmful effects for the child’s health (e.g., tobacco smoking, cannabis use, benzodiazepines and/or antidepressant use, shorter breastfeeding time) [5,8].

Interestingly, in the same environment, in 2004 we reported on a hidden prenatal chronic exposure to cocaine in 4.4% of newborns, together with 5.3% and 8.7% exposure to cannabis and opiates, respectively [6].

Taken together, all these alarming outcomes demonstrate that, when occurring, passive exposure to drugs of abuse is likely maintained throughout childhood, In contrast to tobacco smoking, at present there is not any restrictive legislation on environmental exposure of children to psychoactive drugs. Indeed, whereas the public health system regularly checks the general population and adolescents for consumption of drugs of abuse using self-reported questionnaires surveys [1,2], no screening inquiries or interventions to reduce prenatal or postnatal exposure have been ever put in place.

The present and previous results on this matter let us hypothesizes that these children receive poor familiar attention, in a country like Spain, where drug exposed children are not protected by any specific law promoting foster care or programmed social intervention. Furthermore, it is clear that consumer parents or care givers responsible for their children’s passive exposure are not aware of this risk and possible deleterious health consequences thereof.

## 5. Conclusions

In conclusion, this investigation confirms alarming data about the high passive exposure to drugs of abuse along the last 15 years in infants and children from the Hospital del Mar area of Barcelona, Spain. This occurrence takes place in a disadvantaged socioeconomic environment where consumers are absolutely not aware of the eventual health risks for exposed children. It is likely that this situation can be extrapolated to other similar socioeconomic environments where drugs of abuse consumption by parents and caregivers and more in general by a risky surrounding can lead to unsuspected pediatric passive exposure.

We reaffirm that the use of hair as an alternative matrix to disclose passive exposure to drugs of abuse is an essential tool in risky environments. Public health and social service interventions are needed in order to raise consumer awareness of the risks occurring to children living in the same physical environment where drugs are consumed and to push for avoiding consumption in the places where adults interact with children.
